# Author Correction: Towards critical white ice conditions in lakes under global warming

**DOI:** 10.1038/s41467-023-39005-3

**Published:** 2023-06-06

**Authors:** Gesa A. Weyhenmeyer, Ulrike Obertegger, Hugo Rudebeck, Ellinor Jakobsson, Joachim Jansen, Galina Zdorovennova, Sheel Bansal, Benjamin D. Block, Cayelan C. Carey, Jonathan P. Doubek, Hilary Dugan, Oxana Erina, Irina Fedorova, Janet M. Fischer, Laura Grinberga, Hans-Peter Grossart, Külli Kangur, Lesley B. Knoll, Alo Laas, Fabio Lepori, Jacob Meier, Nikolai Palshin, Mark Peternell, Merja Pulkkanen, James A. Rusak, Sapna Sharma, Danielle Wain, Roman Zdorovennov

**Affiliations:** 1 Department of Ecology and Genetics/Limnology, Uppsala University, Uppsala, Sweden; 2 Fondazione Edmund Mach, Research and Innovation Centre, San Michele all’Adige, Italy; 3 Northern Water Problems Institute, Karelian Research Centre RAS, Petrozavodsk, Russia; 4 U.S. Geological Survey, Northern Prairie Wildlife Research Center, Jamestown, ND USA; 5 Center for Ecological Sciences, Tetra Tech, Inc., Montpelier, VT USA; 6 Department of Biological Sciences, Virginia Tech, Blacksburg, VA USA; 7 School of Natural Resources & Environment, Lake Superior State University, Sault Sainte Marie, MI USA; 8 Center for Freshwater Research and Education, Lake Superior State University, Sault Sainte Marie, MI USA; 9 Center for Limnology, University of Wisconsin-Madison, Madison, WI USA; 10 Lomonosov Moscow State University, Moscow, Russia; 11 St Petersburg State University, St Petersburg, Russia; 12 Department of Biology, Franklin & Marshall College, Lancaster, PA USA; 13 Laboratory of Hydrobiology, Institute of Biology, University of Latvia, Riga, Latvia; 14 Department Plankton and Microbial Ecology, Leibniz Institute for Freshwater Ecology and Inland Fisheries, Stechlin, Germany; 15 Department of Biochemistry and Biology, Potsdam University, Potsdam, Germany; 16 Centre for Limnology, Chair of Hydrobiology and Fishery, Institute of Agricultural and Environmental Sciences, Estonian University of Life- Sciences, Tartu, Estonia; 17 Itasca Biological Station, University of Minnesota, Lake Itasca, MN USA; 18 Institute of Earth Sciences, University of Applied Sciences and Arts of Southern Switzerland, Mendrisio, Switzerland; 19 Department of Earth Sciences, University of Gothenburg, Gothenburg, Sweden; 20 Finnish Environment Institute, Jyväskylä, Finland; 21 Department of Biology, Queen’s University, Kingston, ON Canada; 22 Dorset Environmental Science Centre, Ontario Ministry of the Environment, Conservation and Parks, Dorset, ON Canada; 23 Department of Biology, York University, Toronto, ON Canada; 24 7 Lakes Alliance, Belgrade Lakes, ME USA

**Keywords:** Limnology, Cryospheric science, Environmental impact

Correction to: *Nature Communications* 10.1038/s41467-022-32633-1, published online 25 August 2022

The original version of this Article contained an error in the Results section ‘White ice and ice stability’, which incorrectly read

‘where P is the allowable load in kg, A is the bearing strength that varies between 3.5 and 30 kg cm^−2^ depending on ice quality (30 kg cm^−2^ for cold, black ice conditions of high quality, i.e., no snow ice, no cracks, no bubbles etc. and 3.5 kg cm^−2^ for low quality ice^16^), and H is the total ice thickness in cm.’

The correct version replaces this sentence with

‘where P is the allowable load in kg, A is the bearing strength that varies between 3.5 and 17.5 kg cm^−2^ depending on ice quality (17.5 kg cm^−2^ for cold, black ice conditions of high quality, i.e., no snow ice, no cracks, no bubbles etc. and 3.5 kg cm^−2^ for low quality ice, based on the conclusion of Gold^15^), and H is the total ice thickness in cm.’

The original version of this Article contained an error in in the caption of Figure 3, which incorrectly read

‘Pure black ice conditions were modeled using Eq. (1) with A = 30 kg cm^−2^ and pure white ice conditions using Eq. (2) with A = 3.5 kgcm^−2^’

The correct version replaces this sentence with

‘Pure black ice conditions were modeled using Eq. (1) with A = 17.5 kg cm^−2^ and pure white ice conditions using Eq. (2) with A = 3.5 kgcm^−2^’

This has been corrected in both the PDF and HTML versions of the Article.

