# Supplementary material for: Towards critical white ice conditions in lakes under global warming
Source: Nat Commun. 2022 Aug 25;13:4974. doi: 10.1038/s41467-022-32633-1 (PMC9411540; doi:10.1038/s41467-022-32633-1)
Supplement: Supplementary file 1 — Supplementary Information [file 41467_2022_32633_MOESM1_ESM.pdf]

Supplementary Information

Description of the IceBlitz sampling campaign for participants

Understanding spatial and temporal variation in lake ice properties with help of a global field campaign (IceBlitz)

<u>Main aim:</u> To get a mechanistic understanding of spatial and temporal variation in lake ice thickness, crystal structure and impurities

This Global Lake Ecological Observatory Network (GLEON) project was originally discussed during the GLEON 21 meeting in Canada in the winter limnology working group in autumn 2019. It has the potential to bring us a major step forward towards a better understanding of the challenges and opportunities that a less frozen future will have on nature and society by performing detailed ice property studies. Since lake ice property studies are still comparatively rare, in particular across larger scales, our global approach will open up for the detection of yet unknown ice property changes in a warmer world that are of highest relevance to ice stability and primary production. The global IceBlitz campaign will be complemented by more sophisticated ice property analyses for Swedish lake ice cores in a specialized ice laboratory.

<u>Theory and Implementation:</u> Only a few observations are available on lake ice crystal structure and impurities. According to Michel and Ramseier (1971) three layers can usually be distinguished in lake ice which differ in their crystal structure and character of impurities: primary, secondary, and superimposed ice. Primary ice is the first stage of ice formation. Under calm conditions, thin crystal platelets and needles form on the surface and join together, and a thin ice cover (millimeters) grows as congelation ice horizontally with vertical optical axes (c-axes) of crystals. In cases of wind or snowfall, the surface layer is disturbed, and small frazil crystals (1 mm or less) form and move freely in the turbulent water. Once the buoyancy of the frazil crystals overcomes the turbulence, a solid ice sheet is formed. The optical axes of such ice crystals are randomly oriented. The secondary ice layer grows down from the primary ice layer, and is normally clear (appears as black) congelation ice, which consists of large crystals. The superimposed ice layer forms above the primary ice layer. It is usually snow-ice formed from slush, which is a mixture of snow and liquid water, available from flooding, liquid precipitation or melted snow. Snow-ice grows from the topmost slush down, and then pockets of slush can be captured inside the ice sheet and persist for several weeks. Water on ice (liquid precipitation or meltwater) may also freeze to form superimposed ice. The crystals are as small as those in frazil ice. In the field, the three different ice layers can roughly be distinguished as (from top downwards): 1. Snow-ice (superimposed ice; this layer is often difficult to distinguish from the primary ice layer; see figure below); 2. white ice (corresponds to primary ice), 3. clear (often named as black) ice (corresponds to secondary ice). See figure below where two distinguished layers can be observed and measured.

Here some basic terminology which can be found in the literature:

- Upper ice layer (which correspond to both the superimposed and the primary ice layer described above): snow-ice, frozen slush, white ice, superimposed ice, primary ice, white ice
- Lower ice layer: clear ice, black ice, congelation ice

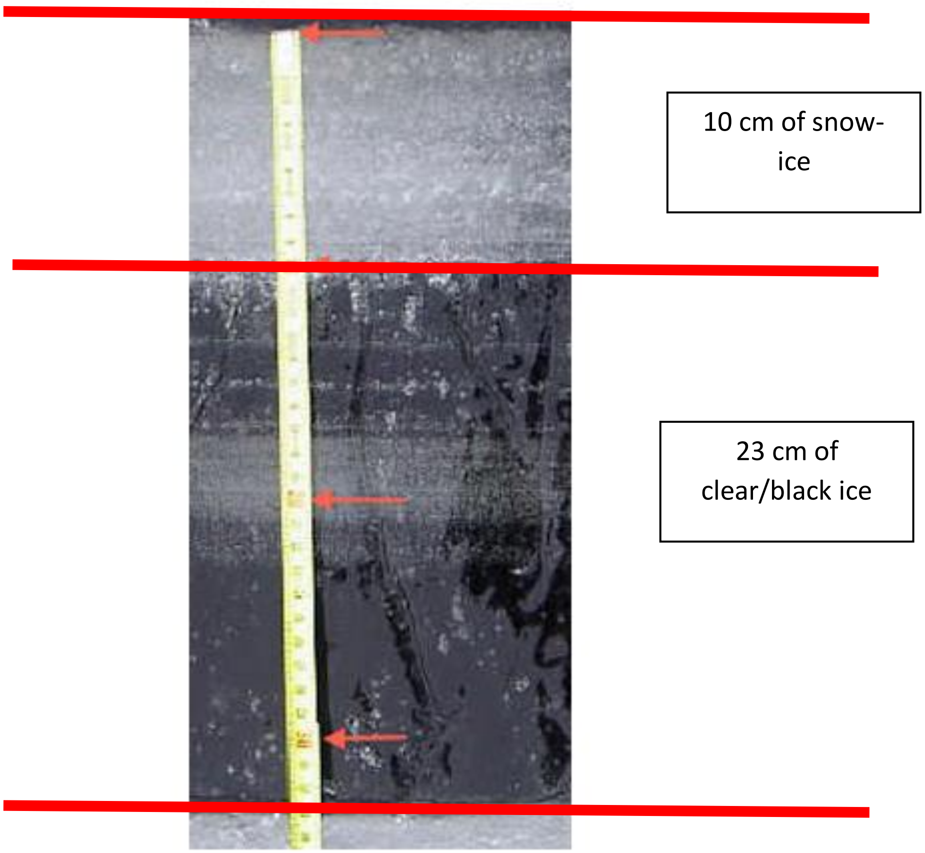

Fig. 2 Stratigraphy of lake ice shown on a photograph of a thick section of ice sheet, Lake Pääjärvi, April 2004. Thickness is 33 cm, composed of 10 cm snow-ice and 23 cm congelation ice. Gas bubble layers are seen in the congelation ice

Picture has been taken from Kirillin et al. (2012)

IceBlitz campaign: Minimum sampling requirements

1. Register the dates of ice on and ice off: the date of ice-on is defined when the main lake area (at least 2/3 of the lake area) is first covered by ice and the ice cover lasts for at least 3 days. The date of ice-off is defined when the main lake area (at least 2/3 of the lake area) is ice-free, implying that some small bays may still have an ice cover. When the lake freezes again, the last date when it opens is taken as the ice-off date.
2. Go out on the ice at least once during winter time and perform the following measurements, preferably at 3 or even 5 different locations of the lake so that we get an idea about spatial variation (if you have a very remote lake, 1 site is also ok) (for more information see excel file which should be used for reporting your measurements):
    - height of snow cover on ice if there is a snow cover
    - total ice thickness,
    - thickness of white ice corresponding to snow-ice
    - thickness of black ice
    - observations of bubbles or other impurities like e.g. dust in the ice
    - water temperature

- air temperature
- take a photo of the ice-covered lake
- if possible, take even a photo from an ice core or the ice layer from the drilled hole

3. In the best case, you repeat this procedure every second week or month so that we can follow the growth of ice over the season. The repetition needs, however, only be done at one single sampling site.

Ice protocol (provided as Excelfile):

Lake name and country	Date of sampling	Coordinates of sampling point and short desciption (i.e. middle of lake, close to shore..)	Height of snow on ice (in cm)	Height of slush on ice (in cm)	Total ice thickness without the snow/slush on ice (cm)	Thickness of white ice, i.e. the upper ice layer (in cm)	Thickness of black ice, i.e. the lower ice layer (in cm)	Are gas bubbles visible in the ice (yes/no)	Are other impurities like e.g. dust visible in the ice (yes/no)	Did you take an ice core (yes/no)	Photo of ice core available (yes/no)	Water temperature at the surface of the drilled hole (°C)	Air temperature 1.5m above drilled hole (°C)	Short description of weather conditions (cloudy, sunny)	Any other ice observations which you would like to mention

References

- Kirillin, G. *et al.* Physics of seasonally ice-covered lakes: a review. *Aquatic Sciences* **74**, 659-682, doi:10.1007/s00027-012-0279-y (2012).
- Michel, B. & Ramseier, R. Classification of river and lake ice. *Can. Geotech. J.* **8**, 36-45 (1971).
